# The Effects of Local Administration of Aminophylline on Transureteral Lithotripsy

**DOI:** 10.1155/2012/727843

**Published:** 2012-10-02

**Authors:** Ayyoub Barzegarnezhad, Abolfazl Firouzian, Seyed Abdollah Emadi, Nadali Mousanejad, Roksana Bakhshali

**Affiliations:** ^1^Department of Urology, Faculty of Medicine, Mazandaran University of Medical Sciences, Sari, Iran; ^2^Department of Anesthesiology, Faculty of Medicine, Mazandaran University of Medical Science, Sari, Iran; ^3^Student Research Committee, Faculty of Medicine, Mazandaran University of Medical Science, Sari, Iran

## Abstract

*Introduction*. Urinary stone is a common cause of urinary tract disease. Stone excretion using ureteroscope is effective in inferior ureter. The aim of this study was to investigate the effects of aminophylline on ureteral spasm during ureteroscopy in acute phase of renal colic. *Methods*. In this double-blind randomized clinical trial, 120 patients with ureteral stones were enrolled and randomized into two groups. The bladder was drained and then received a 150 mL irrigation solution. Irrigation solution was saline and saline plus 10 mL aminophylline at 250 mg dose for control and case groups, respectively. Ureteroscopy and transureteral lithotripsy (TUL) were performed five minutes after irrigation. *Results*. The mean duration of TUL was 4.2 ± 2.61 min and 8.4 ± 2.9 min for control and case groups, respectively. The successful rate was 95% and 76.1% in case and control groups, respectively. Further extracorporeal shock wave lithotripsy (SWL) was performed in 5% and 30% for patients in case and control groups, respectively. *Conclusion*. Aminophylline facilitated ureteroscopy and increased the success rate in the treatment of renal colic using TUL. No significant complications from post-TUL were observed. Using aminophylline carries several advantages such as reducing procedure duration, decreasing the need for ureteral and double-J catheter, and reducing stone migration to the kidney and use of SWL.

## 1. Introduction

Urinary stone (urolithiasis) is the third common cause of urinary tract disease after urinary infection and renal pathologic features [1]. Ureteral stone mostly results in a pain, which begins in the back and radiates to groin, testis in men and major labia in women [1]. The prevalence of renal lithiasis was reported 10–15% in the United States [2]. Urinary stones lead to urinary retention which is the main reason of renal colic. Renal colic is caused by retraction of urinary collective system or ureter. Symptoms of renal colic depends upon stone location. The ureteroscopy is a widely used urologic procedure and applies in various conditions for diagnosis and treatment of urinary diseases like kidney stones [2]. Stone removal using ureteroscope is effective in inferior parts of a ureter [2]. Two types of washing liquid used during urologic intervention are conductor and nonconductor washing agents. Conductor solutions like saline and ringer lactate are not appropriate in endoelectric surgery. Water and glycine are nonconductor agents [2]. For pain management of renal colic, diclofenac, as well as many other nonsteroidal anti-inflammatory drugs, and antispasmodics such as hyoscine butylbromide and morphine can be used [3]. (Since guide wire and ureteroscope are caused complications during entrance to an acute renal colic ureteral spasm, which can be used agents for relive of spasm and increasing of success of ureteroscopy.) These medications are lidocaine gel, aminophylline, and intravenous Buscopan [4].

Aminophylline is a derivative of theophylline. Both aminophylline and theophylline are methylxanthines and are derived from the group called xanthines. The drug aminophylline differs somewhat in its structure from theophylline in that it contains ethylenediamine, as well as more molecules of water. Aminophylline tends to be a less potent and shorter acting than theophylline. Drugs in the Xanthine group relax smooth muscle particularly bronchial muscle, stimulate cardiac muscle, and the central nervous system and produce diuresis. They also cross the placental barrier. These drugs can be given orally, although absorption may be slow and not as effective as when given parenterally. The methylxanthines are partly metabolized in the liver and excreted in the urine. Aminophylline acts through two mechanisms. Firstly, it is a nonselective inhibitor of phosphodiesterase which increases intracellular cAMP, activates PKA, inhibits TNF alpha and synthesis of leukotriene, and decreases inflammation. Secondly, it is a nonselective antagonist of adenosine receptor. Aminophylline is a known drug and widely used in the treatment of acute phase of renal colic [5]. The aim of this double-blind randomized clinical trial was to evaluate the effect of local administration of aminophylline on transureteral lithotripsy.

## 2. Materials and Methods

This double-blinded randomized clinical trial was registered by the Iranian Registry of Clinical Trials under code IRCT201106146803N1. After obtaining approval from the Ethical Committee at Mazandaran University of Medical Sciences, this clinical trial was carried out at the emergency department of Imam Khomeini Hospital and Tooba's Clinical Center in Mazandaran University of Medical Sciences, Sari, Iran. 120 patients with acute renal colic were enrolled for this study; using a computerized software they were randomly assigned into two groups with an equal number of 60 (group A: case and group B: control). All patients gave informed written consent to participate in this study. Patients with history of renal colic, stone excretion, history of transureteral lithotripsy (TUL), anatomic or functional renal disorders and using NSAIDs, corticosteroids, and opioids were excluded. Patients with renal stones <20 mm in distal one 3rd part of ureter and with ages 15–40 years of old were entered in this study.

Case group received normal saline supplemented with aminophylline 250 mg/10 mL, while control group received just normal saline. The saline preparations were made and code labeled by the study nurse. Both urologist and his assistant were blinded on the type of saline preparations they used; they have recorded only the code of saline solution in a check list. At the end of the study, the codes were opened. For this study the first bladder was emptied by using Foley catheter and then 150 mL of the solution was entered using ureteroscope. Ureteroscopy was performed five minutes after administration of the solution. Transureteral lithotripsy was done with pneumatic swiss lithoclast. We used a ureteral stent when significant edema happened and removed it after 12 hours. D-j stent has been used when stone migration, mucosal injury occurred or stone fragment remained in ureter. Further SWL performed at Tooba ESWL Clinic and D-j stent removed after 2 to 4 weeks after SWL.

TUL duration, success rate in stone excretion, further need to extracorporeal shock wave lithotripsy (SWL), and further need to ureteral stent and double J catheter were compared in two groups. Complications during surgery such as tachycardia, hypertension, ureteral avulsion, and acute pyelonephritis related to TUL were recorded.

### 2.1. Statistical Analysis

Data were reported as mean ± standard deviation (SD). The study groups were compared using the Student's *t*-test, Pearson's correlation coefficient for continuous variables, and the chi-square test (or Fisher's exact test if required) for categorical variables. *P* value of 0.05 or less was considered statistically significant. All the statistical analyses were performed using SPSS version 16 (SPSS Inc, Chicago, IL, USA) for Windows.

## 3. Results

TUL was performed on all study subjects. The mean age of patients in case group (group A) and control group (group B) was 34.8 ± 13.2 years (range: 18–39 years) and 35.4 ± 12.7 years (range: 17–40 years), respectively. No significant difference between the two groups with respect to age and gender was observed. In this study, patients were 80 males and 40 females. The average size of patients' renal stones located in inferior ureter was 5.4 ± 3.1 mm and 5.7 ± 4.02 mm in groups A and B, respectively; the difference was not statistically significant.

The mean duration of TUL was 4.2 ± 2.61 min 8.4 ± 2.9 min for group A and group B, respectively, (*P* < 0.05). The success rate of stone removal was 95% (57/60) for case group compared with 71.6% (43/60) for control group with a significant difference between the two groups (*P* < 0.05). In case group, 3 patients (5%) were required ESWL for stone excretion, compared with 18 patients (30%) in control group (*P* < 0.01) (Table 1).

After transureteral lithotripsy, the patients who needed transient ureteral stent were 8 and 11 for case and control groups, respectively. A significant difference between case and control groups was observed for applying double J catheter (Table 1).

Local administration of aminophylline did not cause any post-TUL complications; there was no significant increase in tachycardia and hypertension in case group as compared to control group (*P* > 0.05). This finding supported our idea that systemic absorbtion of aminophylline did not occur.

## 4. Discussion

In our study, aminophylline was locally administrated during TUL; it was related to benefit response rate. Success rate of stone removal in case group was 95% compared with 71.6% in control group. The postoperative stent was required in only 8 patients of case group compared with 11 in control group. No significant side effects were observed in the patients treated with aminophylline. In our study, only 6 patients in case group (compared to 33 in control group) required double J catheter after TUL; this might be related to antispasm effects of aminophylline on ureter. It is established that pharmacological treatment may affect ureteral movement and treatment of renal colic; it can facilitate retrograde access to the ureter and improvement in cleanup of stone or its parts.

Infusion administration of aminophylline was effective in reducing pain and decreasing the required amount of narcotics in symptomatic urinary stones. Since this drug is safe, cheap, and with low side effects, it can be considered as an acceptable alternative or adjuvant treatment to opioid analgesics in renal colic [6]. Intraluminal usage of pharmacologic agents results in independent effects on ureteral dilation and peristaltism in pigs. Theophylline inhibits ureteral peristaltism and verapamil leads to acute dilation of proximal ureter. Ability to change ureteral diameter or peristaltic activity facilitates ureteroscopy [7].

 Aminophylline was locally administrated in collecting system in patients with restricted access to stone due to ureteral or infundibular spasm and also in patients with the uretero-pelvic spasm that could not be differentiated from stone-related stricture. The published results showed that aminophylline was effective in 2 of 3 patients with calyceal staghorn stone and facilitated differentiation of stone-related stricture from uretero-pelvic spasm [8]. Danuser et al. showed that intravenous phenylephrine increased the frequency and extent of recorded contractions, while isoproterenol and phenoterenol decreased these effects. Meanwhile local administration of isoproterenol and phenoterenol had favorable effect comparable to their systemic administrations [9]. Diazoxide, terbutaline, and ritodrine were found to reduce consistently the rate of ureteric peristalsis in animal model. Ritodrine was the most consistent, having a prolonged effect and reducing the rate of ureteric peristalsis to 50% of the rates observed in control experiments [10]. The role of the autonomic nervous system and of cyclic AMP was studied in the control of ureteral peristalsis in isolated guinea pig ureters. Theophylline induced significant dose-dependent reduction in frequency and amplitude of contractions of the ureter hypertonified with barium chloride. No change in frequency or amplitude of contractions was observed with isoproterenol [11]. David F. et al. showed that aminophylline, methylxanthine, and phosphodiesterase inhibitors can relax smooth muscle in the upper urinary tract. A 3 mL amount of 0.5% aminophylline was applied topically to the intrarenal collecting system of 11 patients in whom access to a stone was limited by ureteral or infundibular spasm (three patients) or in whom spasm of the ureteropelvic junction could not be differentiated from stricture (eight patients). Methylxanthine-induced smooth-muscle relaxation may be of value in differentiating spasm secondary to a stone from ureteral scarring and may improve access to peripherally placed renal stones [12].

In Conclusion, we have observed that aminophylline facilitated ureteroscopy and increased the success rate in treatment of renal colic using transureteral lithotripsy. Local administration of aminophylline has several advantages such as short duration of procedure, decreasing a need for double J catheter and ureteral catheter, reducing in-stone migration to the kidney, and decreasing the requirement of extracorporeal shock wave lithotripsy (SWL) in patients. With regards to benefitial effects of local administration of aminophylline, it can be recommended for ureteroscopy in patients.

## Figures and Tables

**Table 1:** Comparison of patients in two groups after TUL.

Variables	Group A (Case)	Group B (control)	*P* value
Duration of TUL (min)	4.2 ± 2.61	8.4 ± 2.9	0.01
Success rate of TUL	57 (95%)	43 (71.6%)	0.071
Further SWL	3 (5.0%)	18 (30.0%)	0.001
Ureteral stent	8 (13.35)	11 (18.3%)	0.071
D-J catheter	6 (10.0%)	33 (55.05)	0.0012
